# Why has Nature Chosen Lutein and Zeaxanthin to Protect the Retina?

**DOI:** 10.4172/2155-9570.1000326

**Published:** 2014-02-21

**Authors:** Justyna Widomska, Witold K Subczynski

**Affiliations:** 1 Department of Biophysics, Medical University of Lublin, Lublin, Poland; 2 Department of Biophysics, Medical College of Wisconsin, Milwaukee, WI, USA

**Keywords:** Macular xanthophylls, Carotenoid, Lutein, Zeaxanthin, Membrane domain, Lipid bilayer, AMD

## Abstract

Age-related macular degeneration (AMD) is associated with a low level of macular carotenoids in the eye retina. Only two carotenoids, namely lutein and zeaxanthin are selectively accumulated in the human eye retina from blood plasma where more than twenty other carotenoids are available. The third carotenoid which is found in the human retina, meso-zeaxanthin is formed directly in the retina from lutein. All these carotenoids, named also macular xanthophylls, play key roles in eye health and retinal disease. Macular xanthophylls are thought to combat light-induced damage mediated by reactive oxygen species by absorbing the most damaging incoming wavelength of light prior to the formation of reactive oxygen species (a function expected of carotenoids in nerve fibers) and by chemically and physically quenching reactive oxygen species once they are formed (a function expected of carotenoids in photoreceptor outer segments). There are two major hypotheses about the precise location of macular xanthophylls in the nerve fiber layer of photoreceptor axons and in photoreceptor outer segments. According to the first, macular xanthophylls transversely incorporate in the lipid-bilayer portion of membranes of the human retina. According to the second, macular xanthophylls are protein-bound by membrane-associated, xanthophyll-binding proteins. In this review we indicate specific properties of macular xanthophylls that could help explain their selective accumulation in the primate retina with special attention paid to xanthophyll-membrane interactions.

## Introduction

**Figure 1:**
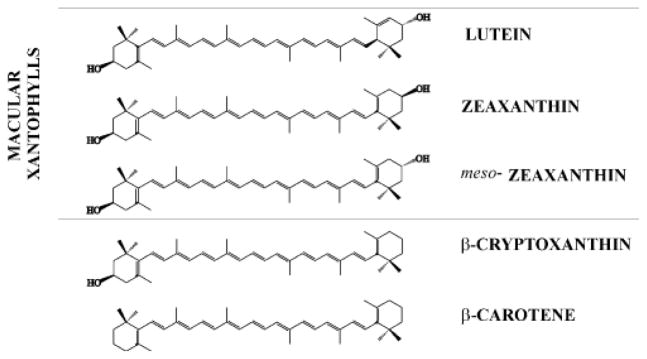
Chemical structures of macular xanthophylls: lutein, zeaxanthin, *meso*-zeaxanthin and two dietary carotenoids: non-polar β-carotene and mono-polar β-cryptoxanthin.

Carotenoids form a group of more than 750 naturally occurring organic pigments [1], only 40 of which are present in the typical human diet [2], and about 20 of them have been detected in human plasma and tissues [3]. Carotenoids can be divided into two main classes: carotenes and xanthophylls. Carotenes are non-polar molecules, which contain only carbon and hydrogen atoms and xanthophylls are polar carotenoids, which contain at least one oxygen atom. In addition, xanthophylls can be subdivided into hydroxyl-carotenoids containing one or two hydroxyl groups and keto-carotenoids containing ketone groups. Unexpectedly, only two carotenoids, namely lutein and zeaxanthin (Figure 1), are selectively accumulated in the membranes of retina from blood plasma, where more than 20 other carotenoids are available. Another carotenoid, meso-zeaxathin (which is a stereoisomer of zeaxanthin, (Figure 1)), is converted from lutein within the retina [4]. The position of the double bond in one of the rings in lutein and zeaxanthin molecules creates differences in retinal distribution of these two pigments in the retina. Zeaxanthin dominates the center region, whereas lutein is dominant in the peripheral region of the retina [5]. In human retina, the concentration of carotenoids reaches a level between 0.1 and 1 mM in the central fovea [6,7], which is about 1000 times higher than in other tissues. Both xanthophylls are accumulated in the region of photoreceptor axons [7] and within photoreceptor outer segments (POSs) [8,9]. Although, macular xanthophylls in POS constitute about 10 to 25% of the amount in the entire retina [8,9], the local concentration of macular xanthophylls in membranes of the rod outer segment is ~70% higher than in residual retina membranes [9]. Moreover, Muller cells have also been suggested as a place for xanthophylls accumulation [10].

The selective uptake of macular xanthophylls into the retina suggests involvement of xanthophyll-binding proteins. It is not clear whether macular xanthophylls are transversely incorporated in the lipid-bilayer portion of retina membranes, or are bound by membrane-associated xanthophyll-binding proteins. Some of the xanthophyll-binding proteins have already been identified and characterized including; GSTP1 (glutathione S-transferases), zeaxanthin-binding protein [11] and 3 StARD3 (steroidogenic acute regulatory domain protein3), lutein-binding protein [12]. The question is whether these proteins are only selective transporters of macular xanthophylls, or whether they are proteins that can store xanthophylls. There is also a significant question of whether the amount of these proteins is sufficient to bind and to store all xanthophyll molecules, which accumulate in the retina in extremely high concentrations. Both interactions of lutein and zeaxanthin with lipid-bilayer membranes and specific proteins are significant. However, in this review, we will focus on xanthophyll membrane interactions.

Age-related macular degeneration (AMD) is multifactorial and a complex disease. However, ageing and oxidative stress seem to be major determinants in pathogenesis. Many epidemiological studies suggest that the higher consumption of lutein and zeaxanthin is associated with lower risk of AMD [13–15]. Such a protective role is attributed to an action of these xanthophylls as antioxidants. There is a huge literature reporting that carotenoids protect phospholipids from peroxidation and that they are efficient singlet oxygen quenchers [16–21]. Why has nature chosen only lutein and zeaxanthin from other carotenoids to protect retina? Chemically they are very similar. The ability of these xanthophylls to filter out blue light (all carotenoids absorb blue/green light) [22] and to quench singlet oxygen (in organic solution the quenching rate constant depends on the number of conjugated double bonds) is not better than that of other plasma carotenoids [21,23]. Therefore, it must be some specific property or properties of these xanthophylls that could help explain their selective presence in the retina. One such property is their disposition and behavior in membranes [24–27]. There are suggestions in the literature that the segregation of polar and non-polar carotenoids already occurs on the level of carotenoid transport. Non-polar carotenoids are transported in human blood plasma primarily in low-density lipoproteins (LDLs), whereas more polar carotenoids are more evenly distributed between LDLs and high-density lipoproteins (HDLs) [28,29]. It is thought that most tissues obtain carotenoids via the LDL receptor route [29]. However, in the case of lutein and zeaxanthin transport, we believe that receptors for HDL should be involved instead. It has been suggested that this role can be played by receptors that are similar to those found in the central nervous system for HDL particles containing ApoE [28,30]. In this review we consider other factors which distinguish lutein and zeaxanthin from other carotenoids in their protective actions against light stress and oxidation in lipid membranes of the retina.

## Xanthophyll-membrane Interactions

The protective function of macular xanthophylls is highly correlated to their membrane localization, in particular, with their membrane solubility, orientation, and distribution between membrane domains. Below, we will present data which support our hypothesis that the high solubility, specific orientation within the lipid bilayer, and the unique lateral distribution between membrane domains of macular xanthophylls maximize their protective action in the eye retina.

### Solubility

Macular xanthophylls are well soluble in lipid bilayers. The reported xanthophyll solubility thresholds (concentration of xanthophylls at which aggregation initiates) in fluid-phase model membranes lie in the area of 10 mol% for zeaxanthin and 15% for lutein [31], but values as high as 17 mol% [32] and 28 mol% [33] have been reported. A lower value such as 5 mol% was also reported for zeaxanthin incorporated into unilamellar vesicle formed with dipalmitoylphosphatidylcholine [34]. Non-polar β-carotene starts to aggregate at a concentration as low as 0.5 mol% [35]. Mono-polar β-cryptoxanthin is also less soluble in the lipid bilayer than macular xanthophylls [27]. Interestingly, the tendency of cis-isomers of xanthophylls to aggregate is usually much less than their all-trans counterparts [36,37], and they also affect membrane properties more strongly [38]. Based on the solubility measurements, we can make a first conclusion: *the high membrane solubility of macular xanthophylls is one of the major characteristics that distinguish them from other dietary carotenoids*.

There are two problems which we would like to mention here. The first is dealing with terminology. In earlier papers the terms “solubility” and “incorporation” were often used interchangeably. In our discussion we use the term “solubility” to refer to the amount of carotenoids dissolved in the lipid bilayer as monomers, while the term “incorporation” refers to the amount of carotenoids present in the lipid bilayer in the form of monomers, dimers, oligomers and aggregates. Socaciu et al. [39] measured the incorporation ratio of different carotenoids in different membranes. They found high incorporation for xanthophylls and low incorporation for β-carotene. The second problem is dealing with the membrane preparation. To the best of our knowledge, in most, if not all papers, the solubility and aggregation of carotenoids were investigated in membranes prepared using the film deposition method [40] or some variations of this method. During membrane preparations using the film deposition method, the lipid mixture passed through the solid-state intermediate at which solid-state demixing of carotenoids can occur. Carotenoid molecules, which can be trapped in carotenoid aggregates during this preparation, do not participate in further liposome formation producing false estimates of carotenoid solubility thresholds. This can explain large scattering of data about carotenoids solubility in lipid bilayers. This problem was faced during measurements of cholesterol solubility in model membranes and was solved with the new method of membrane preparation, namely the rapid solvent exchange method [41,42].

### Transmembrane localization

The transmembrane localization of a significant portion of macular xanthophylls in retinal cells seems to be obvious [43–48]. The presence of polar hydroxyl groups at the ends of macular xanthophyll molecules (Figure 1) ensures their perpendicular or close to perpendicular orientation in the bilayer. Non-polar carotenoids are oriented rather randomly [49]. Macular xanthophylls with this transmembrane orientation and high membrane solubility affect membrane properties strongly. In particular, they decrease membrane fluidity [27,45,46,50], especially decreasing strongly the frequency of vertical fluctuations of the terminal methyl groups of alkyl chains toward the membrane surface [51]. At a high concentration, macular xanthophylls induce formation of the liquid-ordered phase in model membranes [50], an action similar to that caused by cholesterol [52]. They also cause a considerable increase of hydrophobicity of the membrane interior [53], which strongly affects ion penetration into the membrane. Most significantly macular xanthophylls reduce the oxygen concentration and oxygen diffusion at all locations in the membrane [50,54]. This effect will be discussed in Section: Oxygen concentration and diffusion.

The changes in the membrane properties should affect chemical reactions occurring within the lipid bilayer. Often these changes make membranes less sensitive to oxidative damage (these problems are discussed in a review [55] and in Section: Xanthophylls impede light-induced damage to retina). The transmembrane localization of macular xanthophylls in retinal membranes can also explain their very slow removal from the retina, observed after discontinuation of xanthophyll supplements given to healthy volunteers [56]. These observations suggest that anchoring xanthophyll molecules at opposite membrane surfaces is significant not only in enhancing their effects on membrane properties [27,47,48], but also in stabilization of these molecules in membranes of the human retina. Thus, we can summarize: *transmembrane orientation of macular xanthophylls distinguishes them from other dietary carotenoids, enhances their stability in retina membranes, and maximizes their protective action in the eye retina.*

### Distribution between membrane domains

In the above sections we emphasized effects of membrane modifiers (macular xanthophylls) on membrane properties. These effects can change drastically with membrane composition and depth in the lipid bilayer [27]. Also the membrane itself, especially membrane lateral organization into membrane domains, can affect lateral distribution of membrane modifiers, including macular xanthophylls. It has been shown that in membranes of retinal pigment epithelium and photoreceptors, raft domains are present [57–60]. Raft domains have been postulated to enhance signal transduction [61–63], and are also involved in lipid sorting [64] and protein trafficking/recycling [59,65]. Rafts in membranes of photoreceptor cells are involved in regulation of the G-protein-mediated pathway of photo-transduction [59]. Aggregation of small, unstable rafts in bigger platforms (observed, for example, in retinal pigment epithelium cells) is supposed to enhance signal transduction to the cell interior and cause a specific reaction in the cell, such as apoptosis [66].

**Figure 2:**
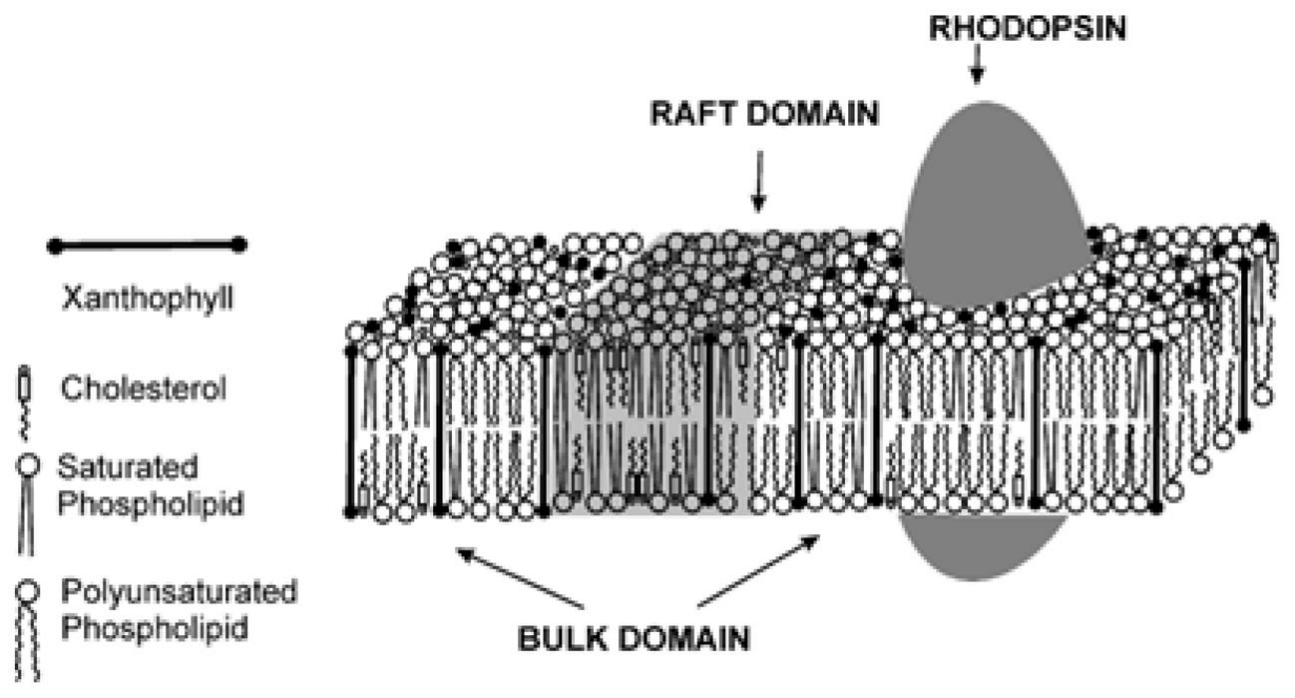
Schematic drawing showing the distribution of macular xanthophylls between the saturated raft domain and the unsaturated bulk domain in membranes of POSs. Rhodopsin is also included to show its co-localization with unsaturated lipids and xanthophylls.

Raft domains in photoreceptor outer segment (POS) disc membranes are surrounded by the bulk lipid domain rich in long chain polyunsaturated fatty acids including docosahexaenoic acid (DHA) with a six double-bond chain [8,67,68]. Also, rhodopsin, which is the main protein of POS membranes and is responsible for the first stages of visual signal transduction, is located in the bulk domain of the POS membrane [57,58,68,69]. Rhodopsin requires the presence of polyunsaturated lipids (DHA) for its activity [70–72], and thus their co-localization is functionally justified. In the model of POS membranes, macular xanthophylls were about 14 times more concentrated in the bulk domain (enriched in polyunsaturated DHA) and were substantially excluded from the raft domain (enriched in saturated lipids and cholesterol) [26] (Figure 2). This unique distribution was confirmed in membranes made of a raft-forming mixture where macular xanthophylls were about eight times more concentrated in the bulk domain than in the raft domain [73]. A similar distribution has been observed for mono-polar xanthophyll, namely β-cryptoxanthin. However, non-polar β-carotene was more uniformly distributed between domains [24]. These results strongly support statements that in POS membranes macular xanthophylls will also be concentrated in the bulk domain and excluded from the raft domain. Such a selective accumulation of macular xanthophylls in domains rich in vulnerable unsaturated lipids seems to be ideal for their antioxidant action [24]. Thus, we can hypothesize that co-localization of macular xanthophylls, polyunsaturated phospholipids, and rhodopsin in POS membranes (Figure 2) may enhance the antioxidant action of xanthophylls. This hypothesis was confirmed by experiments in which the protective role of lutein against lipid peroxidation in membranes made of raft forming mixtures and in models of POS membranes was compared to lutein antioxidant action in homogenous membranes composed of unsaturated lipids [74].

**Figure 3:**
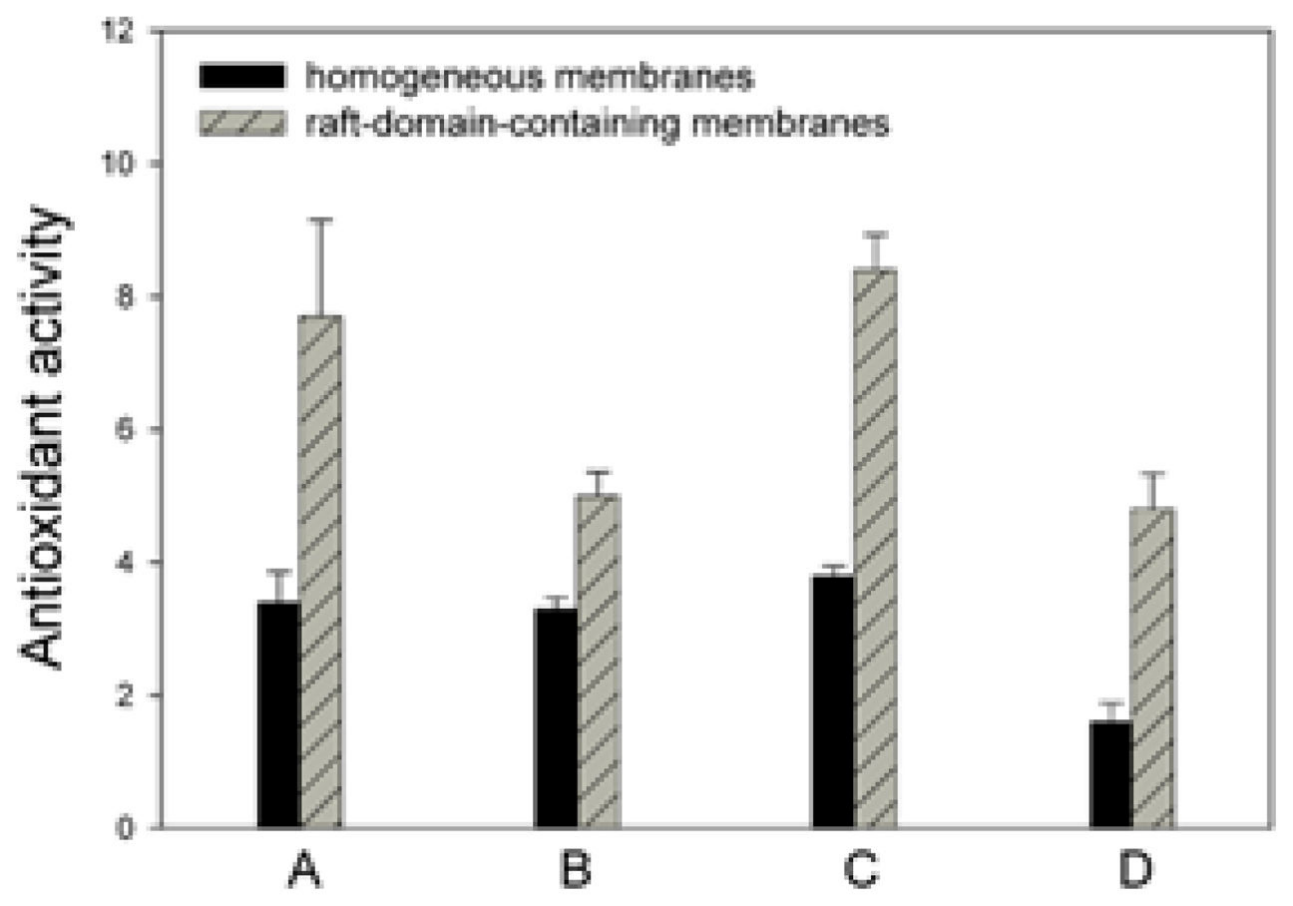
Comparison of the antioxidant activity of the macular xanthophyll, lutein, in raft-domain-containing and homogeneous membranes. Antioxidant activity is expressed as (A) a ratio of the rate of lipid hydroperoxide accumulation in membranes in the absence and presence of 0.1 mol% lutein, as a ratio of the oxygen consumption rate in membrane suspension in the absence and presence of (B) 0.3 mol% and (C) 0.5 mol% lutein, and (D) as a ratio of the MDA-TBA adduct accumulation rate in the absence and presence of 0.5 mol% lutein. Homogeneous membranes were made of dioleoylphospatidylcholine (DOPC) (A, B, and C) and didocosahexaenoylphosphatidylcholine (DHAPC) (D). Raft-domain-containing membranes were made of DOPC/sphingomyelin/cholesterol equimolar mixtre (A, B, and C) and DHAPC/distearoylphosphatidylcholine/cholesterol equimolar mixture (D). For more details see Ref. [74].

The rate of lipid peroxidation was inhibited in the presence of lutein, and inhibition was significantly greater in membranes containing raft domains than in homogenous membranes (Figure 3). We can conclude: *the domain structure allows location of macular xanthophylls in the most vulnerable regions of POS membranes. This localization is ideal if macular xanthophylls are to act as a lipid antioxidant, which is the most accepted mechanism through which lutein and zeaxanthin protect the retina from AMD* [75–78].

## Xanthophylls Impede Light-induced Damage to Retina

### Light absorption

There are two main functional explanations for the selective presence of lutein and zeaxanthin in the retina. One is the necessity of photoprotection against the oxidative stress and macular xanthophylls serve that role very well. Another functional hypothesis is based on the fact that the pigments are localized mostly in the outer plexiform also known as Henle’s layer [43] and therefore form a filter for blue light. Most ultraviolet below 300 nm is absorbed by cornea [78], whereas ultraviolet in range 300–400 nm is blocked by the lens. Light transmission by the lens decreases with ageing, particularly at shorter wavelengths [79]. Nevertheless, some fraction of blue radiation reaches the retina and may activate potent photosensitizers retinal photosensitizers such as all-trans retinal, cytochrome c oxidase, porphyrins [80–83], and consequently generates reactive species. *Macular xanthophylls, due to their appropriate location, may significantly reduce macular blue-light toxicity on the retina and improve protection against oxidative damage. Blue-light absorption can be considered an indirect antioxidant action because it prevents blue light from generating reactive oxygen species that can damage photoreceptor cells* [84].

**Figure 4:**
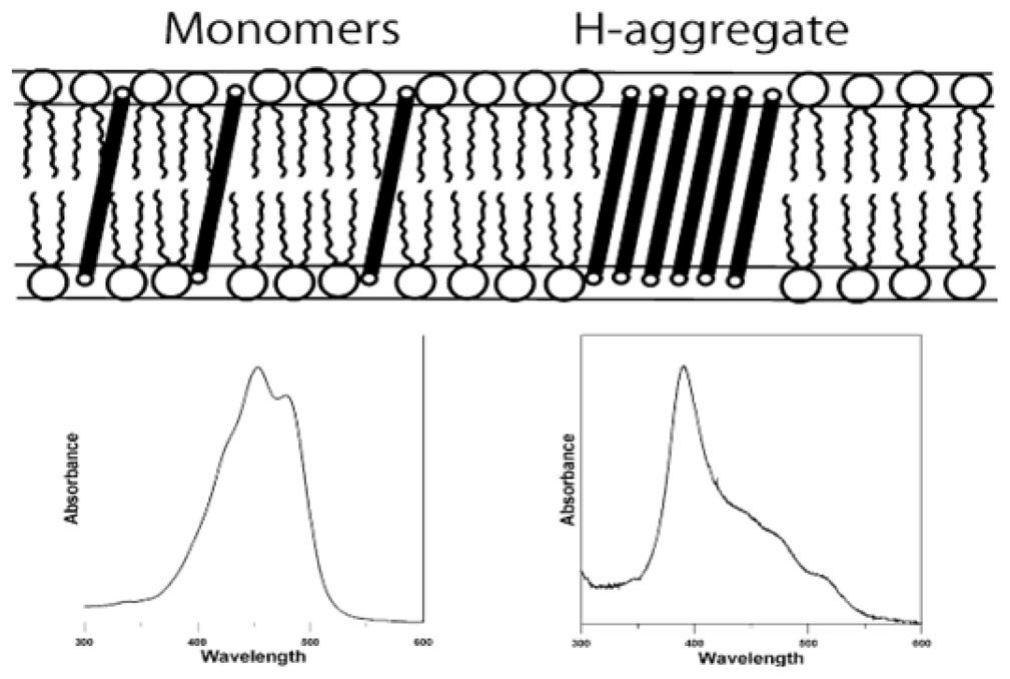
Schematic drawing of the location of macular xanthophylls in the lipid bilayer membrane. Monomers and H-aggregate are indicated together with their absorption spectra.

It is well known that carotenoids in form of monomers absorb light in range 390 nm-540 nm with maximum absorption in the region of 450 nm, whereas in form of aggregates maximum absorption may be shifted to lower wavelength. In the case of “card-pack” arrangement (H-aggregates) the shift to the shorter wavelength is observed (blue shift). In the case of “head-to-tail” organization (J-aggregates) the shift to the longer wavelength is observed (red shift). In lipid bilayers macular xanthophylls can be present as monomers or can form H-aggregates with blue-shifted absorption spectrum (Figure 4). Junghans et al. [85] has investigated the blue-light filter efficiency of four plasma carotenoids (lutein, zeaxanthin, β-carotene, and lycopene) incorporated into membranes of liposomes loaded with the hydrophilic fluorescent dye, Lucifer yellow, excitable by blue light. Fluorescent emission of the dye was lower in liposomes with carotenoids as compared to the control, indicating filter effect. Macular xanthophylls zeaxanthin and lutein exhibited the highest blue-light absorption activity as compared with liposomes containing non-polar carotenoids, β-carotene, and lycopene.

Blue-light absorption by macular xanthophylls is extremely important for young eyes, for which the lens transparency is almost 95%. During aging the lens gradually loses its transparency, become yellowish [79], and better filtrate UV and blue light. Thus, in older age the blue-light filtration performed by macular xanthophylls becomes relatively less important.

Macular xanthophylls may not only act as a blue-light filter, but also optimize visual performance. The layer of macular xanthophylls is believed to reduce chromatic aberrations, glare disability, and light scattering which enhance vision contrast [86].

### Physical quenching of reactive oxygen and photosensitizers

Carotenoids have been known to be the most effective singlet oxygen quenchers and their activities are much higher than that of another retinal antioxidant pigment α-tocopherol [18,87]. They are able to quench singlet oxygen by two different mechanisms. The first mechanism, which involves energy transfer, termed physical quenching, is considered the major pathway of singlet oxygen deactivation. According to this mechanism carotenoid molecules deactivate singlet oxygen to the nonreactive triplet state. During that process carotenoid molecules become excited to the triplet state and can return to the ground state dissipating the energy excess as heat. The profit of the physical quenching is that carotenoids may act without alternation of their own chemical structure. The second mechanism is called chemical quenching. It involves a chemical reaction between carotenoid and singlet oxygen which results in pigment autooxiadation. The capacity of major plasma carotenoids to quench singlet oxygen in an organic solvent mainly depends on the number of conjugated double bonds in the chromophore, but also varies with functional groups [23]. Thus, zeaxanthin (11 conjugated double bonds) has a higher ability to quench singlet oxygen than lutein (10 conjugated double bonds) (Figure 1).

Macular xanthophylls may quench singlet oxygen directly because their triplet energy level is lower than the energy level of singlet oxygen. They are also capable of quenching excited triplet states of potent singlet oxygen photosensitizers. That property is well known as non-photochemical quenching in plants. By this mechanism the largest part of excess energy is transferred from potentially harmful chlorophyll triplets to lutein and dissipated as heat [88,89]. Similarly, photoactivation of rhodopsin (also located in the unsaturated bulk domain, see Section: Distribution between membrane domains) leads to isomerization of its chromophore, 11-cis-retinal to all-trans-retinal, which under certain conditions can act as a photosensitizer. Free all-trans retinal may absorb light and transfer energy from its excited triplet state to molecular oxygen, generating singlet oxygen [90]. Close proximity of xanthophylls, which are also located in the bulk domain, allow effective energy transfer from excited all-trans-retinal to xanthophyll and prevent singlet oxygen generation by this photosensitizer [91]. We would like to summarize this section indicating: *the membrane domain structure plays a significant role in the enhancing protection of retina against oxidative damage through the co-localization of macular xanthophylls with harmful molecules (singlet oxygen) and harmful processes (formation of singlet oxygen and formation of photosensitizers).*

### Chemical antioxidant action

Carotenoids effectively quench singlet oxygen through physical quenching (see Section: Physical quenching of reactive oxygen and photosensitizers). However, inactivation of that harmful molecule may also occur through chemical quenching involving carotenoids autoxidation. This latter process consumes carotenoids themselves. Chemical quenching contributes less than 0.05 % to the overall singlet oxygen quenching by carotenoids [92]. The degradation of four major plasma carotenoids, induced by UV light in presence of rose Bengal, has been studied [93,94]. The higher degradation rates were found for non-polar carotenoids as compare to macular xathophylls. Also studies of the autooxidation of carotenoids incorporated in pig liver microsomes [95] give similar results, non-polar carotenoids such as β-carotene and lycopene had degraded totally, whereas the degradation of polar carotenoids was much slower, and zeaxanthin was shown to be the most stable carotenoid. We can conclude: *high chemical stability of macular xanthophylls distinguish them from other dietary carotenoids*.

The conjugated double bond system is primarily responsible for high chemical reactivity of carotenoids with both singlet oxygen [23,87] and free radicals [96]. Selective localization of macular xanthophylls in domains rich in polyunsaturated phospholipids (see Section: Distribution between membrane domains), and therefore susceptible to a free radical-induced damage, is ideal for their chemical antioxidant action. Carotenoids scavenge lipid peroxyl radicals by forming radical adducts [96] which are less reactive than lipid alkyl peroxyl radicals. Thus, carotenoids are effective chain-breaking antioxidants, which delay the oxidation of bio-membranes by trapping the chain-initiating or chain-propagating peroxyl radicals.

Interesting conclusions, which fit to the goal of this review, can be made by comparison of the antioxidant properties of macular xanthophylls with antioxidant properties of other dietary carotenoids investigated in organic solvents and in lipid bilayer membranes. For example, zeaxanthin and non-polar β-carotene show similar antioxidant properties in organic solutions. However, their antioxidant properties differ when incorporated into membranes [97]. Zeaxanthin was shown to react with free radicals slightly more effectively than β-cryptoxanthin and much more effectively than β-carotene [98,99]. β-Carotene and lycopene are able to react efficiently only with radicals generated inside the membrane. Macular xanthophylls, with their hydroxyl groups exposed to an aqueous environment, can also scavenge free radicals generated in the aqueous phase [37].

Antioxidant activity of carotenoids can be related to their effects on physical properties of lipid bilayer membranes [100]. Strong ordering effect of the dipolar xanthophyll, astaxanthin, is accompanied by its strong antioxidant activity. Non-polar carotenoids like β-carotene and lycopene, which disorder the membrane, acted as relatively poorer antioxidants than the xanthophylls. β-Carotene, because of its low membrane solubility as a monomer and low incorporation efficiency, and, therefore, weak effects on membranes does not protect membranes against lipid peroxidation. Although at oxygen tensions close to 1 atm it may act as a prooxidant [101]. However, these conditions are not relevant for retina and other human tissues and organs. Above examples allow us to conclude: *the presence of polar hydroxyl groups at the ends of macular xanthophylls and their transmembrane orientation enhance their antioxidant properties, as compared with the antioxidant properties of other dietary carotenoids.*

### Oxygen concentration and diffusion

The microenvironment in which membrane-located reagents are immersed can change drastically with membrane composition and depth in the lipid bilayer [55]. Oxygen is involved in most important damaging chemical reactions within the membrane which include lipid peroxidation and the formation of reactive oxygen species and the oxidative damage is postulated to be a major cause of AMD [102]. Additionally, antioxidant action of macular xanthophylls is mainly confined to the membrane environment. All these indicate that the effect of the microenvironment on the local oxygen concentration and local oxygen diffusion coefficient should modulate damaging and protective reactions involved in AMD. Thus, knowledge of profiles of oxygen concentration and oxygen diffusion across membranes or membrane domains is extremely important.

One of the authors (WKS) was involved in the development of a spin-label oximetry method which allows measurement of the oxygen diffusion-concentration product in restricted domains such as membranes, or more accurately, at a certain depth in membranes [103,104]. This product is of fundamental interest for understanding chemical reactions involving oxygen, and separation into its component factors (the diffusion coefficient and concentration) is not necessary. These results are usually presented as profiles of the oxygen-diffusion concentration product across membranes. Membrane modifiers affect profiles of this product differently in different membrane regions and membrane domains. For example, cholesterol significantly decreases the oxygen diffusion-concentration product in the polar head group region and in the hydrocarbon region near polar head groups, and increases it in the membrane center [52,105,106] (Figure 4A). Macular xanthophylls decrease the oxygen diffusion-concentration product in saturated and unsaturated membranes [54,27]. The effect is strongest in the membrane center and negligible in the head group region. At 10 mol% macular xanthophylls decrease the diffusion-concentration product in the center of lipid bilayer membranes by 30% (Figure 4B).

**Figure 5:**
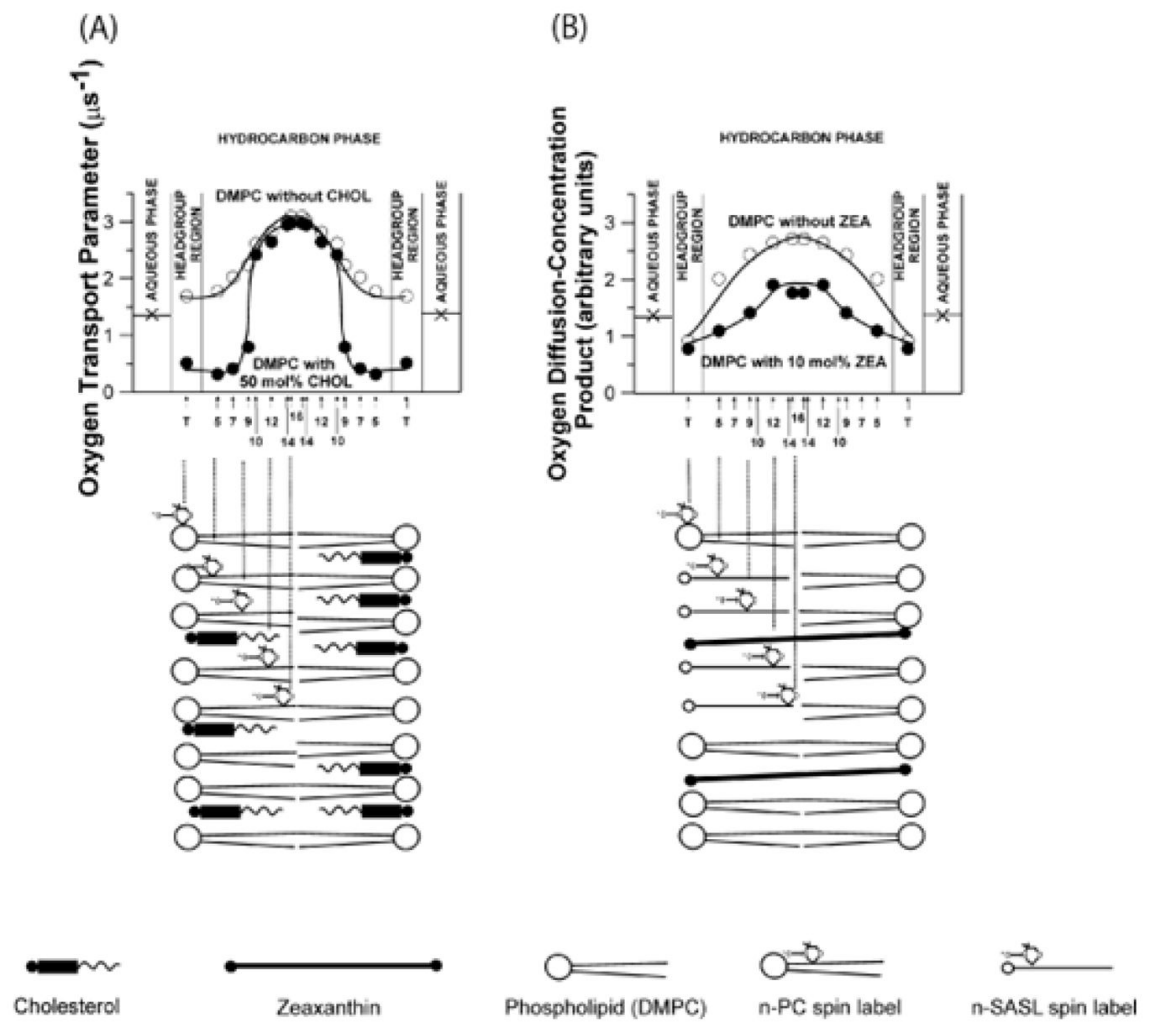
Profiles of the oxygen diffusion-concentration product across the dimyristoylphosphatidylcholine membrane measured at 25°C in the absence (○) and presence (●) of 50 mol% cholesterol (A) and 10 mol% zeaxanthin (B). Measurements in (A) were done using saturation-recovery EPR approach with phospholipid-type spin labels. Measurements in (B) were done using line-broadening EPR approach with stearic acid spin labels (SASLs). Approximate locations of the nitroxide moieties of spin labels are indicated by arrows. The nitroxide attached to C16 may pass through the center of the bilayer and stay in the other leaflet of the membrane. Schematic drawings indicate relative positions of membrane modifiers (cholesterol and zeaxanthin) in the lipid bilayer. Figure was made based on data presented in [52] and [54].

The effect of carotenoids on the oxygen-diffusion concentration product can have physiological significance for organisms with a high carotenoid content in their membranes, for example, for bacteria, and in some situations, for plants, in which the local carotenoid concentration in the lipid bilayer can reach a value up to a few mol%. In animals, the highest carotenoid concentration is found in the eye retina of primates, but even here the carotenoid concentration in the lipid-bilayer portion of the membrane is much lower than 1 mol% [43]. Figure 5, however, illustrates profiles of the oxygen diffusion-concentration product in membranes in the presence of different membrane modifiers. The different effects of cholesterol and polar carotenoids on oxygen transport can result from different structures and different localization of these molecules in the membrane. The cholesterol molecule is located in one half of the bilayer, and its rigid plate-like portion extends to the depth of the 7th to 10th carbon atoms in lipid hydrocarbon chains [107]. In contrast, one carotenoid molecule influences both halves of the lipid bilayer and, with two polar groups interacting with opposite hydrophilic surfaces of the membrane; it can brace together the two halves of the bilayer like a tie-bar [108]. Therefore, the oxygen diffusion-concentration product is reduced in those regions of the bilayer to which the rigid portion of the molecule of the modifier extends (see schemes in Figure 4).

In retina membranes macular xanthophylls are mainly located in the bulk membrane domain with the profile of the oxygen diffusion-concentration product similar to upper profile in Figure 4A. They are substantially excluded from the raft domain with the profile of the oxygen diffusion-concentration product similar to lower profile in Figure 4A. Oxygen environment is very different in both situations. Thus, macular xanthophylls are localized in the membrane domain which is extremely susceptible to lipid peroxidation not only because it is rich in polyunsaturated DHA but also because the oxygen diffusion concentration product is two to four times greater in the bulk domain than in the raft domain [26], which makes polyunsaturated lipids located in the former domain even more susceptible to oxidative damage.

Generation of singlet oxygen straightforwardly depends on the local oxygen-diffusion-concentration product. A good example related to the xanthophyll protection of the retina membranes is the generation of singlet oxygen by the photosentitizer, all-trans-retinal (as described in Section: Physical quenching of reactive oxygen and photosensitizers). High oxygen diffusion-concentration product in the bulk domain of POS membranes should increase photoproduction of singlet oxygen during collisions of molecular oxygen with all-trans-retinal. The presence of macular xanthophylls in this, highly oxygenated domain, reduces oxidative damage. We can conclude: *the membrane domain structure plays a significant role in the protection of retina membranes against oxidative damage through the co-localization of potentially harmful molecules (molecular oxygen) with protective molecules.*

## Concluding Remarks

**Figure 6:**
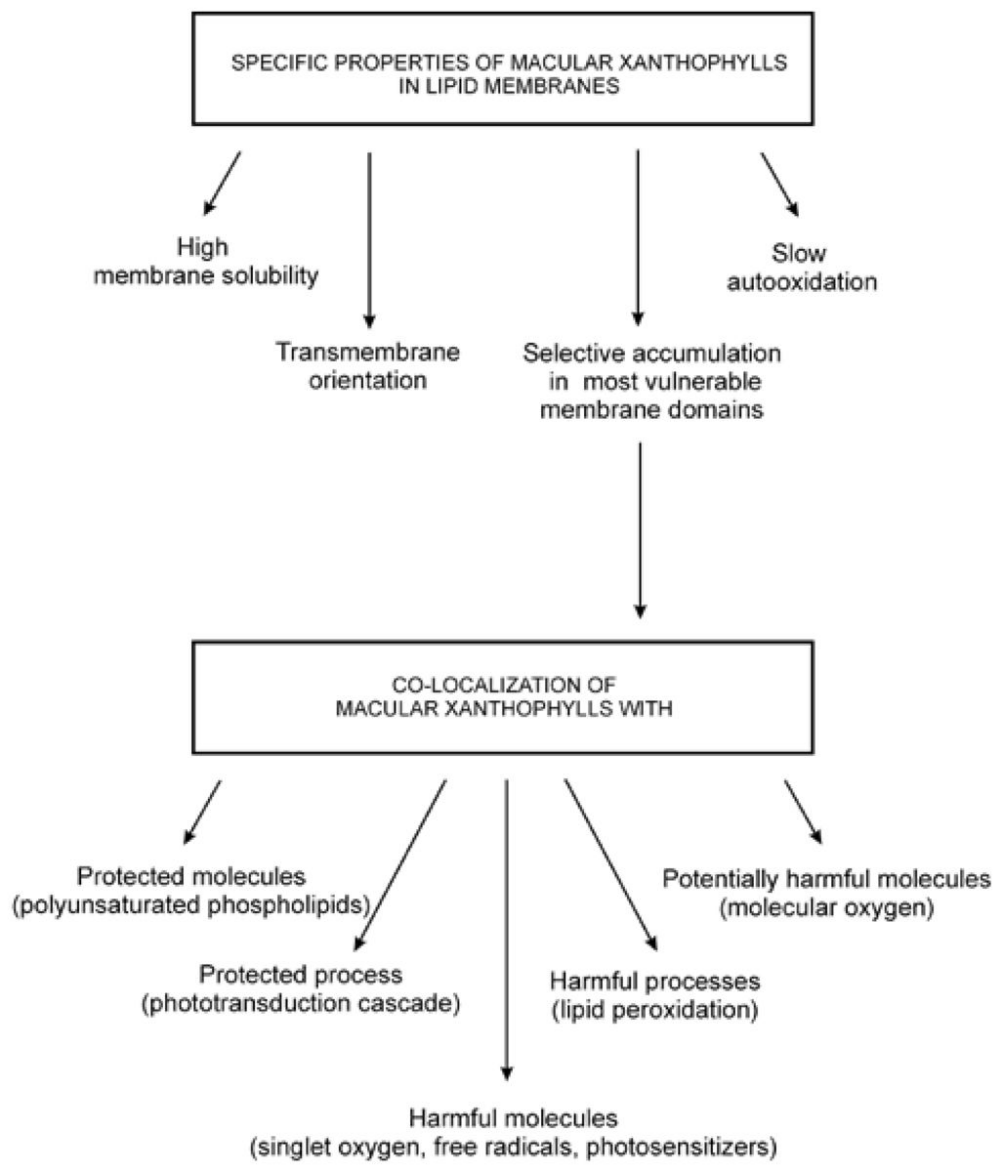
Diagram indicating how the lipid bilayer membrane and its domain structure affect the organization of macular xanthophylls within the membrane and how this xanthophyll organization affects their protective activity in membranes of POSs.

The diagram in Figure 6 summarizes our conclusions indicating major macular xanthophyll-membrane interactions, which can modulate or enhance their protective antioxidant actions in retina. Macular xanthophylls are highly soluble in lipid membranes with the preferential transmembrane orientation. These ensure both, physical and chemical stability of these carotenoids in the retina. Physical stability is manifested by their very slow removal from the retina, observed after discontinuation of xanthophyll supplementation.

Macular xanthophylls, when located in lipid bylayer membranes, are also degraded more slowly than other dietary carotenoids, thus are chemically more stable. We think, however, that the most significant consequence of macular xanthophyll-membrane interaction is their selective accumulation in the bulk domain of the POS membrane. Rhodopsin is also located in the bulk domain of the POS membrane. Additionally, this domain is enriched in long-chain polyunsaturated phospholipids (C18-C24), as well as in very-long-chain polyunsaturated phospholipids (> C24) with 3–9 double bonds [109,110]. It has been suggested that very-long-chain polyunsaturated phospholipids likely play a unique, important role in the retina because they are necessary for cell survival and their loss leads to cell death [111,112]. It has been also suggested that they are tight-bound to rhodopsin, and that their unusually long chains may partially surround the α-helical segments of rhodopsin [113]. Co-localization of rhodopsin with polyunsaturated phospholipids creates a dangerous situation for both, especially during illumination when reactive oxygen species can be produced by photosensitizers. To protect the retina against oxidative damage, nature has used xanthophylls as an effective protector that can absorb damaging blue light, neutralize photosensitizers and reactive oxygen species, and scavenge free radicals. Co-localization of protective and protected molecules should significantly enhance the effectiveness of protectors, especially when the local concentration of xanthophylls in the membrane is not very high.
